# Major adverse cardiovascular events are associated with necroptosis during severe COVID-19

**DOI:** 10.1186/s13054-023-04423-8

**Published:** 2023-04-20

**Authors:** Rosana Wiscovitch-Russo, Elsa D. Ibáñez-Prada, Cristian C. Serrano-Mayorga, Benjamin L. Sievers, Maeve A. Engelbride, Surya Padmanabhan, Gene S. Tan, Sanjay Vashee, Ingrid G. Bustos, Carlos Pachecho, Lina Mendez, Peter H. Dube, Harinder Singh, Luis Felipe Reyes, Norberto Gonzalez-Juarbe

**Affiliations:** 1 Infectious Diseases and Genomic Medicine Group, J Craig Venter Institute, 9605 Medical Center Drive Suite 150, Rockville, MD 20850 USA; 2 Unisabana Center for Translational Science, Universidad de La Sabana, Chía, Colombia; 3 Clinica Universidad de La Sabana, Chía, Colombia; 4 Division of Infectious Diseases, Department of Medicine, University of California San Diego, La Jolla, CA 92037 USA; 5 Department of Microbiology, Immunology and Molecular Genetics, The University of Texas Health Science Center at San Antonio, San Antonio, TX 78229 USA; 6 Pandemic Science Institute, University of Oxford, Oxford, UK; 7 Present Address: Boehringer Ingelheim, Ames, IA USA

**Keywords:** COVID-19, Necroptosis, Heart injury

## Abstract

**Background:**

The mechanisms used by SARS-CoV-2 to induce major adverse cardiac events (MACE) are unknown. Thus, we aimed to determine if SARS-CoV-2 can induce necrotic cell death to promote MACE in patients with severe COVID-19.

**Methods:**

This observational prospective cohort study includes experiments with hamsters and human samples from patients with severe COVID-19. Cytokines and serum biomarkers were analysed in human serum. Cardiac transcriptome analyses were performed in hamsters' hearts.

**Results:**

From a cohort of 70 patients, MACE was documented in 26% (18/70). Those who developed MACE had higher Log copies/mL of SARS-CoV-2, troponin-I, and pro-BNP in serum. Also, the elevation of IP-10 and a major decrease in levels of IL-17ɑ, IL-6, and IL-1rɑ were observed. No differences were found in the ability of serum antibodies to neutralise viral spike proteins in pseudoviruses from variants of concern. In hamster models, we found a stark increase in viral titters in the hearts 4 days post-infection. The cardiac transcriptome evaluation resulted in the differential expression of ~ 9% of the total transcripts. Analysis of transcriptional changes in the effectors of necroptosis (mixed lineage kinase domain-like, MLKL) and pyroptosis (gasdermin D) showed necroptosis, but not pyroptosis, to be elevated. An active form of MLKL (phosphorylated MLKL, pMLKL) was elevated in hamster hearts and, most importantly, in the serum of MACE patients.

**Conclusion:**

SARS-CoV-2 identification in the systemic circulation is associated with MACE and necroptosis activity. The increased pMLKL and Troponin-I indicated the occurrence of necroptosis in the heart and suggested necroptosis effectors could serve as biomarkers and/or therapeutic targets.

*Trial registration* Not applicable.

**Supplementary Information:**

The online version contains supplementary material available at 10.1186/s13054-023-04423-8.

## Introduction

COVID-19 has been the most devastating infectious disease since the 1918 influenza pandemic over 100 years ago [1], shaking healthcare systems and rattling the scientific community. It is projected to cause a cumulative worldwide loss of about USD 12.5 trillion and up to 8.76 million lives through 12/2024 [2, 3]. While infection with severe acute respiratory syndrome coronavirus 2 (SARS-CoV-2), the causative agent of COVID-19, is often associated with respiratory and pulmonary-related diseases, it can also lead to detrimental effects on several organs and induce systemic complications. Major adverse cardiac events (MACE) (*i.e*., heart attacks, arrhythmias, heart failure, and strokes) are some of the most frequently diagnosed complications of COVID-19 [4–7]. MACE has been shown to occur during acute hospitalisation and even once the patient is discharged from the hospital, leading to worse clinical outcomes, including higher mortality [8–10]. Despite these observations, the exact mechanisms by which infection with SARS-CoV-2 leads to MACE is unknown.

SARS-CoV-2 uses the angiotensin-converting enzyme 2 (ACE2) as the receptor to enter host cells. ACE2 is an enzyme in the renin-angiotensin system that regulates blood pressure and electrolyte homeostasis [11, 12]. ACE2 expression is highest in the thyroid, heart, kidney, testis, and small intestine [13]. Furthermore, the expression of ACE2 is increased in cardiac tissue upon biological stress and chronic cardiovascular diseases such as heart failure [14] and respiratory infection by *Streptococcus pneumoniae* or influenza A virus [15]. These reports suggest that the myocardium is susceptible to SARS-CoV-2 entry and can potentially lead to the development of MACE via a combination of direct viral infection, circulating Spike protein interaction with ACE2 or systemic inflammation. Programmed cell death can be driven by viral infections and death receptor ligands such as inflammatory cytokines [16] and is a fundamental process in cardiac pathologies. In recent years, multiple studies have described how major forms of cell death modulate cardiac injury, adverse cardiac remodelling, and heart failure [17]. Apoptosis is a quiescent immune form of cell death modulated by a group of cysteine proteases, i.e. caspases [18]. In contrast to apoptosis, forms of programmed necrosis have significant roles in cardiac pathologies due to the promotion of exacerbated inflammation and irreparable tissue damage [19–21]. These are mainly encompassed by pyroptosis, necroptosis, ferroptosis, and mitochondrial-mediated necrosis [22]. Recently, we reported that pandemic influenza virus infection led to cardiac pathogenesis. We observed that necroptosis and mitochondrial damage play a major role in influenza-infected mice and human cardiomyocytes in cardiac damage during influenza infection [23]. Necroptosis is a highly inflammatory form of cell death regulated by the receptor-interacting serine/threonine-protein kinases (RIPK)1 and RIPK3 and the effector molecule mixed lineage kinase domain-like protein (MLKL) [24].

Several studies have documented the development of MACE in patients with COVID-19 [25, 26], mainly those with severe infection, comorbid conditions, and older age. Moreover, some researchers have documented that MACE is also frequent in patients that survive the acute infection and are now recognised as part of the long-COVID syndrome [27]. However, the underlying mechanisms of these complications still need to be better understood. Several studies have shown the presence of SARS-CoV-2 viral particles in the bloodstream and in the cardiac tissue of patients that succumbed to infection [25, 28–31]. Recent reports also showed that SARS-CoV-2 could infect human cardiomyocytes via ACE2, replicate, and cause cell death in vitro [32, 33]. However, it is unknown whether SARS-CoV-2 presence in the bloodstream or its ability to infect cardiomyocytes is associated with the tissue injury and cell death that promotes the development of MACE. In addition, it is uncertain whether serum biomarkers could be used to identify patients at higher risk of developing MACE during acute COVID-19. Here we attempted to bridge these gaps to identify potential therapeutic targets to prevent MACE in patients infected with SARS-CoV-2.

## Materials and methods

This observational prospective cohort study includes experiments with hamsters, and human samples gathered from subjects admitted to the Clínica Universidad de La Sabana in Chía, Colombia, with confirmed COVID-19 diagnosed by reverse transcription polymerase chain reaction (RT-PCR). All consecutive patients admitted with severe disease to the participating centre were included between November 2019 and May 2020. Data were collected prospectively by the attending physicians by reviewing medical records, laboratory data, and blood samples within the first 24 h of hospital admission were gathered to dissect the underlying mechanisms of MACE in these patients. This study was approved by the Institutional Review Board (IRB) of the Clínica Universidad de La Sabana, and all patients signed informed consent to participate in the study (CUS-LFR-012).

### Subjects and data collection

The human cohort includes hospitalised patients older than 18 years. Disease severity was defined based on the World Health Organisation criteria. Severe illness was diagnosed in patients with SpO^2^ ≤ 94% on room air, including patients on supplemental oxygen, oxygen through a high-flow device, or no-invasive ventilation. Critical illness was diagnosed in patients requiring invasive mechanical ventilation and/or extracorporeal membrane oxygenation or end-organ dysfunction.

During hospital admission, the following variables were collected: demographic data, comorbidities, symptoms, physiological variables collected during the first 24 h of hospital admission, systemic complications, and laboratory reports. A retrospective chart review was conducted at hospital discharge to double-check the registered data.

### Study definitions

MACE is a composite outcome [34, 35] encompassing patients who develop the following clinical diagnoses. *Cardiac arrhythmia* (new or worsening): change from the sequence of electrical impulses in the electrocardiogram (EKG), compared to EKG at hospital admission or in past medical history [36]. *Heart Failure* (new or worsening): a clinical syndrome with symptoms and/or signs secondary to functional or structural cardiac abnormality, which may occur with or without previous cardiac disease documented through an echocardiogram, evidence of pulmonary or systemic congestion, and/or an increase in serum biomarkers such as Pro-BNP [37]. *Myocardial injury*: acute cardiac cell injury corroborated by the rise of serum troponin values with at least one value above the 99th percentile of the normal reference value of each local laboratory; development of pathological Q waves and/or new ischemic changes in EKG; evidence of coronary thrombus by angiography and/or new loss of viable myocardium, or regional wall motion abnormality identified in the echocardiogram [38, 39]. Finally, *stroke* was defined as a neurological deficit caused by an acute focal injury of the central nervous system by a vascular cause (i.e. cerebral infarction, intracerebral haemorrhage, or subarachnoid haemorrhage) [40].

### Virus strain and hamster infection

SARS-CoV-2 isolate USA-WA-1/2020 was used for these studies. The reagent was deposited by the Centres for Disease Control and Prevention and obtained through BEI Resources, NIAID, NIH: SARS-Related Coronavirus 2, Isolate USA-WA-1/2020, NR-52281. Male golden Syrian hamsters of 3–4 weeks old were purchased from Charles River Laboratory and used at 5–6 weeks of age. All procedures were per approved IACUC (2020040AR and 2020048AR) and Institutional Biosafety Committee protocols (08,496). Hamsters were infected with the indicated dose of virus intratracheally (i.t.) as previously described (31); animals were sedated with ketamine (100 mg/kg) and xylazine (5 mg/kg), and the tongue was pulled forward to visualise the trachea. Three hundred microliters of the virus at a dose of 9 × 10^5^ PFU of SARS-CoV-2, USA-WA-1/2020 suspension was applied to the trachea, and then the nose was covered to stimulate respiration.

### Testing

All the specific performed testing, such as the quantification of cytokines and chemokines, RNA sequencing, neutralisation assays, and western blots, are explained in the Additional file 2.

### Statistical analysis

Categorical variables are presented in counts (percentages) and were evaluated through the Chi-square test or Fisher's exact test. Continuous variables with normal distribution are expressed as means (standard deviation); variables with no normal distribution are expressed as median (interquartile ranges). For continuous variables with normal distribution, the t-Student test was performed, and for variables with no normal distribution, the Wilcoxon-Mann–Whitney test was used. Descriptive and bivariate analysis of the information was performed to determine the association between inflammatory profile and clinical outcomes, such as in-hospital mortality, the requirement of invasive mechanical ventilation, ICU admission, and hospital length of stay.

### Data sharing

The raw RNA-seq data have been deposited to the NCBI database under the accession number Bio project ID–PRJNA884511.

## Results

**Fig. 1:**
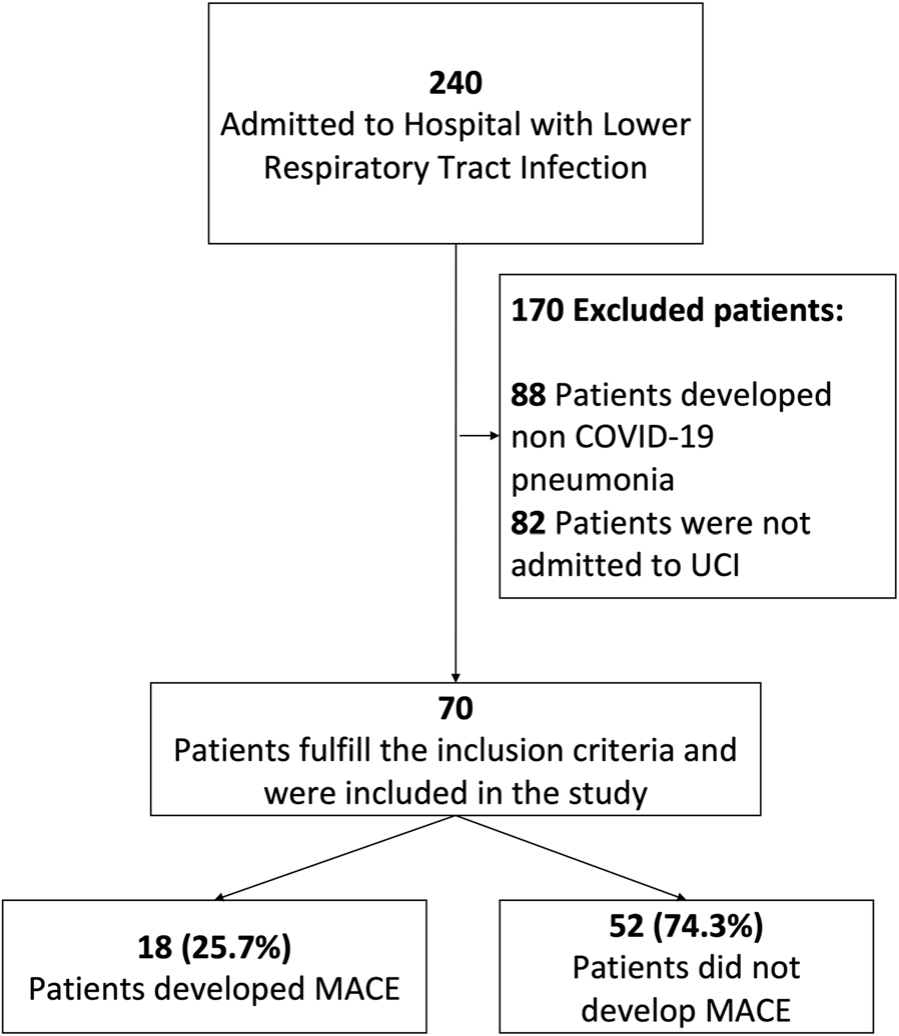
Flow chart and diagnoses. Flow chart of patient included in the study

A total of 70 patients with confirmed SARS-CoV-2 infection admitted to the intensive care unit (ICU) were included in the study (Fig. 1). The median (IQR) age was 61.5 (50.5–69.0), and most of the patients were males (80% [56/70]). The main comorbidities were arterial hypertension (58.6% [41/70]), obesity (18.6% [13/70]), and diabetes mellitus (17.1% [12/70]) (Table 1). More than a quarter of patients developed MACE during ICU stay (25.7% [18/70]), and a total of 22 MACE were reported. The most frequent MACE diagnoses in the cohort were myocardial injury (50% [9/18]), followed by new onset arrhythmia (27.8% [5/18]), worsening heart failure (22.2% [4/18]), and myocardial infarction (11.1% [2/18]). Interestingly, only one case of new-onset cardiac failure (5.6% [1/18]) and cardiovascular death (5.6% [1/18]) were documented.

**Table 1:** Demographics characteristics of patients hospitalised due to COVID stratified by the presence of Major Adverse Cardiovascular Events (MACE)

Characteristic	All *n* = 70	MACE *n* = 18	NO-MACE *n* = 52	*P value*
*Demographic*				
Male. *n* (%)	56 (80.0)	18 (100)	38 (73.1)	**0.03**
Age. median (IQR)	61.5 (50.5–69.0)	67.5 (53.3–70.8)	59.0 (49.8–68.0)	0.40
*Comorbid conditions. n (%)*				
Stroke	1 (1.4)	0 (0.0)	1 (1.9)	0.58
Myocardial infarction	4 (5.7)	3 (16.7)	1 (1.9)	0.08
Cardiac arrhythmia	3 (4.3)	3 (16.7)	0 (0.0)	**0.02**
Asthma	1 (1.4)	0 (0.0)	1 (1.0)	0.58
Diabetes mellitus	12 (17.1)	5 (27.8)	7 (13.7)	0.30
Coronary disease	2 (2.9)	1 (5.6)	1 (1.9)	0.98
Mental Illness	1 (1.4)	0 (0.0)	1 (1.9)	0.58
Chronic kidney disease	3 (4.3)	2 (11.1)	1 (1.9)	0.33
COPD	8 (11.4)	1 (5.6)	7 (13.5)	0.63
Congestive cardiac failure	2 (2.9)	2 (11.1)	0 (0.0)	0.11
Arterial hypertension	41 (58.6)	10 (55.6)	31 (59.6)	0.98
Obesity	13 (18.6)	5 (27.8)	8 (15.4)	0.42
OSAHS	4 (5.7)	0 (0.0)	4 (7.7)	0.53
Smoking	9 (12.9)	2 (11.1)	7 (13.5)	0.88
Dyslipidaemia	3 (4.3)	3 (16.7)	0 (0.0)	**0.02**
*Physiological variables during the first 24 h of admission. median (IQR)*				
Heart rate. BPM	93.5 (82.5–111.0)	96.5 (83.3–116.5)	92.0 (83.5–110.3)	0.16
Respiratory rate. BrPM	22.0 (20.0–30.0)	24.0 (20.0–30.0)	22.0 (20.0–27.8)	0.63
Temperature. °C	36.5 (36.3–37.0)	36.7 (36.4–37.1)	36.4 (36.2–37.0)	0.16
SBP. mmHg	120.5 (108.5–131.0)	124.5 (115.5–131.0)	119.5 (104.5–131.0)	0.55
DBP. mmHg	70.0 (64.0–79.0)	70.5 (64.5–75.5)	70.0 (64.0–80.25)	0.63
MAP. mmHg	87.0 (79.5–98.0)	87.5 (83.3–93.0)	86.5 (78.0–98.3)	1.00
SPO2. (%)	87.0 (72.75–91.0)	80.5 (67.8–89.8)	88.5 (80.5–91.0)	0.12
Glasgow	15.0 (15.0–15.0)	15.0 (15.0–15.0)	15.0 (15.0–15.0)	0.18
*Laboratory variables at admission. median (IQR)*				
Presepsin, ng/L	716.0 (471.8–1743.8)	1080.5 (615.8–2458.8)	683.5 (429.3–1596.0)	0.14
Pro-BNP, pg/mL	715.5 (171.5–1443.3)	1860.5 (939.8–4526.5)	398.0 (132.0–1029.5)	** < 0.001**
Troponin, ng/mL	12.9 (4.9–89.7)	106.5 (23.2–354.0)	8.9 (3.7–21.5)	** < 0.001**
D Dimer, mg/mL	1.5 (0.9–3.3)	1.2 (0.7–3.3)	1.6 (1.0–3.2)	0.45
WBC, cell × 103	9.1 (7.1–12.4)	8.6 (7.1–11.1)	9.2 (7.2–13.2)	0.51
Neutrophils, (%)	83.6 (78.4–89.4)	85.4 (78.4–89.7)	83.5 (79.1–89.3)	0.84
Haemoglobin, g/dL	15.8 (14.4–17.5)	16.5 (15.1–17.6)	15.6 (14.3–17.2)	0.42
Platelet, cell × 103	236.5 (179.3–269.3)	226.5 (178.3–263.8)	237.0 (179.8–271.5)	0.73
Creatinine, mg/dL	1.1 (0.8–1.3)	1.1 (1.0–1.8)	1.0 (0.8–1.2)	0.07
BUN, mg/dL	19.9 (15.1–27.3)	23.9 (14.5–29.1)	19.1 (15.1–26.4)	0.34
Blood glucose, mg/dL	138.1 (116.0–163.9)	167.5 (129.5–209.3)	129.9 (114.9–149.0)	**0.02**
Sodium, mEq/L	136.9 (135.0–139.0)	135.0 (133.0–136.8)	137.0 (135.0–140.3)	**0.01**
Potassium, mEq/L	4.3 (4.1–4.6)	4.5 (4.1–5.0)	4.3 (4.0–4.5)	0.10
Chloride, mEq/L	98.6 (94.0–101.9)	96.4 (89.4–99.2)	99.0 (94.6–103.0)	**0.03**
Bilirubin max, mg/dL	0.7 (0.5–1.3)	0.7 (0.5–0.9)	0.8 (0.5–1.4)	0.26
ALT, U/L	60.5 (38.9–83.4)	53.9 (38.9–83.0)	60.9 (40.6–87.5)	0.46
AST, U/L	62.9 (47.7–80.7)	62.8 (48.9–74.9)	62.9 (49.0–83.3)	0.60
pH	7.5 (7.4–7.5)	7.4 (7.4–7.5)	7.5 (7.5–7.5)	**0.02**
PCO2, mmHg	29 (26.3–33)	29.0 (27.0–36.0)	28.0 (26.0–32.0)	0.44
PaO2, mmHg	59 (51–66)	54.0 (45.3–59.8)	61.0 (51.8–67.0)	0.04
HCO3, mmol/L	21.5 (19.2–23.9)	21.6 (17.4–25.3)	21.5 (19.4–23.8)	0.89
Lactic acid, mmol/L	1.6 (1.2–2.1)	1.8 (1.3–2.5)	1.6 (1.2–2.1)	0.33
CRP, mg/L	204.4 (128.7–276.3)	246.1 (174.7–274.7)	191.2 (122.3–274.8)	0.46
PT, seconds	12.6 (11.5–13.4)	12.2 (11.2–12.7)	12.7 (11.9–13.4)	0.24
PTT, seconds	27.4 (26.1–29.8)	28.5 (26.9–31.2)	27.2 (25.9–28.6)	0.11
*Outcomes*				
Hospital LOS, days (IQR)	12.5 (9.0–18.0)	10.5 (9.0–14.5)	14.0 (9.0–19.0)	0.33
In-Hospital Mortality (%)	27 (38.6)	11 (61.1)	16 (30.8)	**0.02**

Bold indicates statistically significant variables

*MACE* Major cardiovascular events, *IQR* Interquartile range, *COPD* Chronic obstructive pulmonary disease, *OSAHS* Obstructive sleep apnoea-hypopnea syndrome, *BPM* Beats per minute, *BrPM* Breaths per minute, *SBP* Systolic blood pressure, *DBP* Diastolic blood pressure, *MAP* Mean arterial pressure, *SPO2* Oxygen saturation, *BNP* Brain natriuretic peptide, *WBC* White blood cell count, *BUN* Blood urea nitrogen, *ALT* Alanine aminotransferase, *AST* Aspartate aminotransferase, *PCO2* Partial pressure of carbon dioxide, *PaO2* Partial pressure of oxygen, *HCO3* Bicarbonate, *CRP* C reactive protein, *PT* Prothrombin Time, *PTT* Thromboplastin time, *LOS* Length of stay

Patients who developed MACE were older than those who did not (median [IQR], 67.5 years old [53.3–70.8] vs 59.0 years old [49.8–68.0], *p* = 0.04). In addition, patients who developed MACE had more frequently a past medical history of cardiovascular diseases, such as myocardial infarction (16.7% [3/18] vs 1.9% [1/52]; *p* = 0.08), cardiac arrhythmia (16.7% [3/18] vs 0.0 [0/52]; *p* = 0.02), dyslipidaemia (16.7 [3/18] vs 0.0 [0/52]; *p* = 0.02), and congestive heart failure (1.1% [2/18] vs 0.0 [0/52]; *p* = 0.11). Both groups (i.e. MACE and no-MACE) had similar physiological variables at admission; however, some laboratory results differed among the groups (Table 1). For instance, chloride, sodium, and pH levels were significantly lower in MACE patients than in no-MACE patients (96.4 mEq/L [89.4–99.2] vs 99.0 mEq/L [94.6–103.0], *p* = 0.03; 135.0 mEq/L [133.0–136.8] vs 137.0 mEq/L [135.0–140.3], *p* = 0.01; 7.4 [7.4–7.5] vs 7.5 [7.5–7.5], *p* = 0.02; respectively) (Table 1).

Regarding the clinical outcomes, MACE patients had a shorter length of stay when compared with no-MACE patients; however, this difference was not statistically significant (10.5 days [9.0–14.5] vs 14.0 days [9.0–19.0], *p* = 0.33). In contrast, we found that patients who developed MACE during hospital admission had a significantly higher mortality rate (61.1% [11/18] vs 30.8% [16/52], *p* < 0.02). All demographic characteristics are presented in Table 1.

### The relation between serum viral load and the development of MACE

**Fig. 2:**
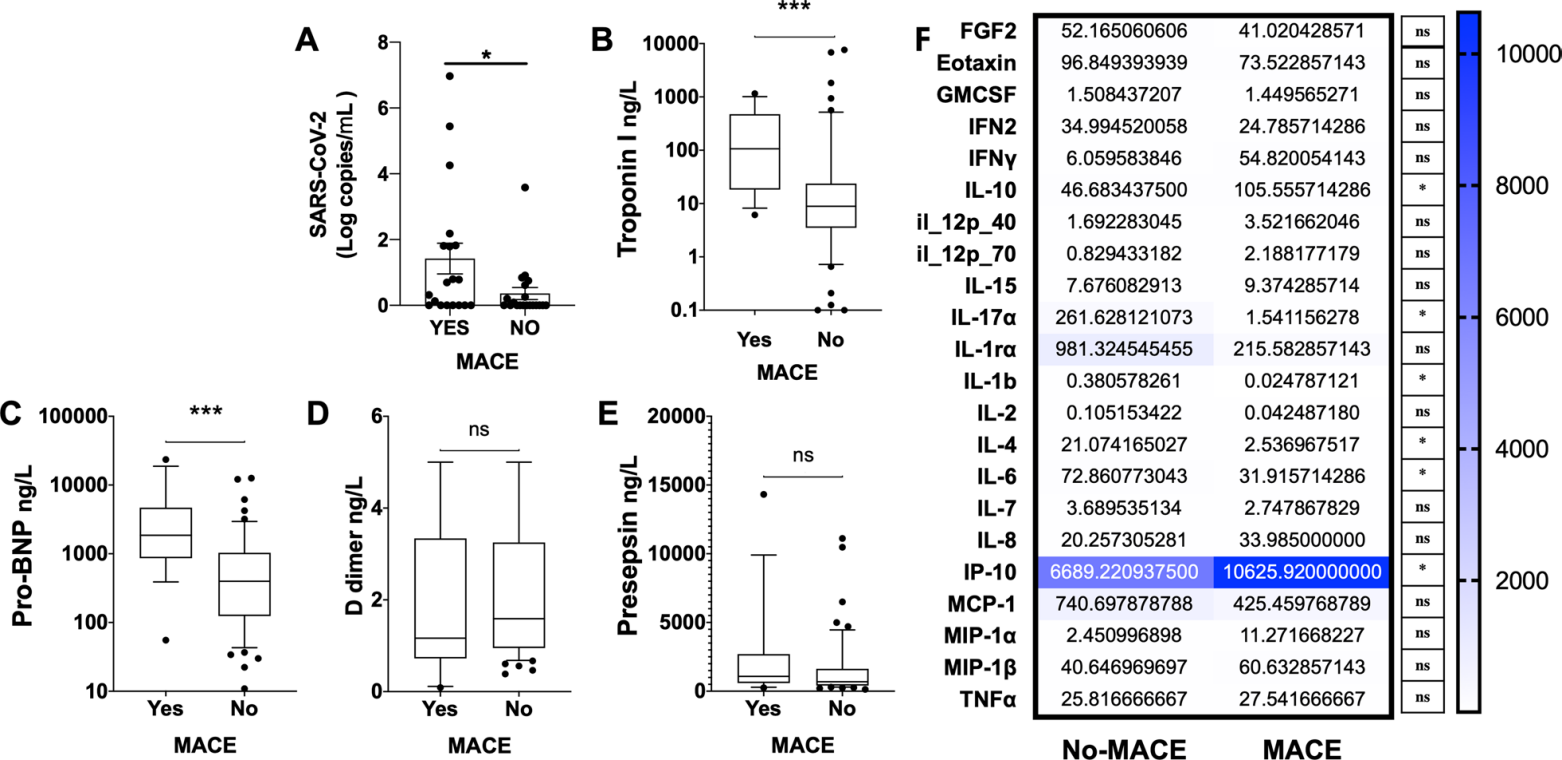
MACE patients increase markers of cardiac injury and SARS-CoV-2 presence. **A** Log copies per mL of SARS-CoV-2 tested via quantitative RT-PCR. Changes in **B** Troponin I, **C** Pro-BNP, **D** D dimer, and **E** Presepsin (ng per L of serum). As measured by multiplex analysis, **F** Cytokine, and chemokine changes in serum (pg per mL). Mean ± SEM analysed by Kruskal–Wallis test with Dunn's multiple-comparison post-test (**A**–**E**), *t*-tests used in F. Asterisks denote the level of significance observed: * = *p* ≤ 0.05; ** = *p* ≤ 0.01; *** = *p* ≤ 0.001

To determine whether serum viral load was associated with the development of MACE, we assessed the amount of SARS-CoV-2 in the serum of the cohort at hospital admission. We found that the patients who developed MACE had higher Log copies/mL of SARS-CoV-2 (1.422 [0.000–6.971] vs 0.363 [0.000–3.582], *p* < 0.05) when tested via quantitative RT-PCR in serum (Fig. 2A).

### The relation of serum biomarkers and cytokines with MACE development

Different critical mediators were measured to define possible biomarkers for MACE development. MACE patients had increased serum concentration of cardiac biomarkers, principally Troponin I (106.5 ng per mL [23.2–354.0] vs 8.9 ng per mL [3.7–21.5]; *p* < 0.001) (Fig. 2B) and pro-B-type natriuretic peptide (pro-BNP) (1860.5 [939.8–4526.5] vs 398.0 [132.0–1029.5]; *p* < 0.001) at hospital admission (Fig. 2C). Notably, D-dimer and presepsin were not increased in MACE patients at hospital admission (Fig. 2D, 2E). Moreover, cytokines levels also showed differences among MACE and no-MACE patients, mainly by elevation of interferon-$\gamma$-induced protein-10 (IP-10) (6689.22 ng per mL [1083.44–20,327.7] vs 10,625.92 ng per mL [3492.59–18,129.20]; *p* = 0.05) and interleukin (IL)-10 (105.56 ng per mL [28.83–243.87] vs 46.68 ng per mL [3.58–271.29]; *p* < 0.05) or a decrease in levels of IL-17ɑ (1.54 ng per mL [0.0000745–7.22] vs 261.63 ng per mL [0.00000411–1822]; *p* < 0.05), IL-1β (0.025 ng per mL [0.00144–0.052] vs 0.381 ng per mL [0.000356–6.68]; *p* < 0.05), IL-4 (2.53 ng per mL [0.412–8.97] vs 21.07 ng per mL [0.006–149.76]; *p* < 0.05), and IL-6 (31.91 ng per mL [16.79–60.21] vs 72.86 ng per mL [0.06–345.13]; *p* < 0.05) compared to the no-MACE group (Fig. 2F).

### Neutralisation of SARS-CoV-2 variants and the development of MACE

**Fig. 3:**
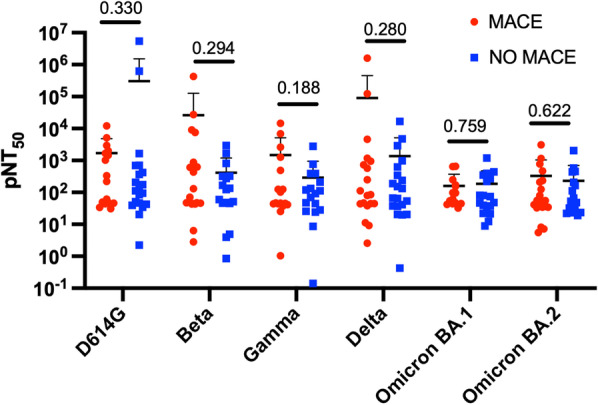
Plasma-neutralising titters in patients with MACE show no significant changes. pNT50 values against the four SARS-CoV-2 pseudo-viral variants Beta, Gamma, Delta, and Omicron BA.1 and BA.2 and the control early 2020 strain with the D614 mutation. They were measured in samples collected from patients experiencing MACE and those with no signs of MACE (No-MACE)

Spike-specific neutralising antibodies are generally considered correlates of protection against COVID-19. We want to determine whether there were any significant differences in the neutralisation titters between the MACE and no-MACE groups, which could explain the disease trajectory following ICU admission. No significant differences were observed in the ability to neutralise pseudoviruses from the variant of concerns: SARS-CoV-2 (D614) or Beta, Gamma, Delta, and Omicron subvariants (both BA.1 and BA.2). While a partial increase in the neutralisation of D614 was observed in the no-MACE group and an increase of Delta neutralisation in the MACE group, these were not statistically significant. Our results suggest that at the time of ICU admission, the robustness of neutralising antibody activity does not appear to be involved in preventing MACE (Fig. 3). Moreover, these data also suggest that MACE was not associated with a particular viral variant of concern.

Hearts of hamsters infected with SARS-COV-2 show upregulation of major pathways associated with injury, cell death, antiviral immune responses, and metabolic changes.

**Fig. 4:**
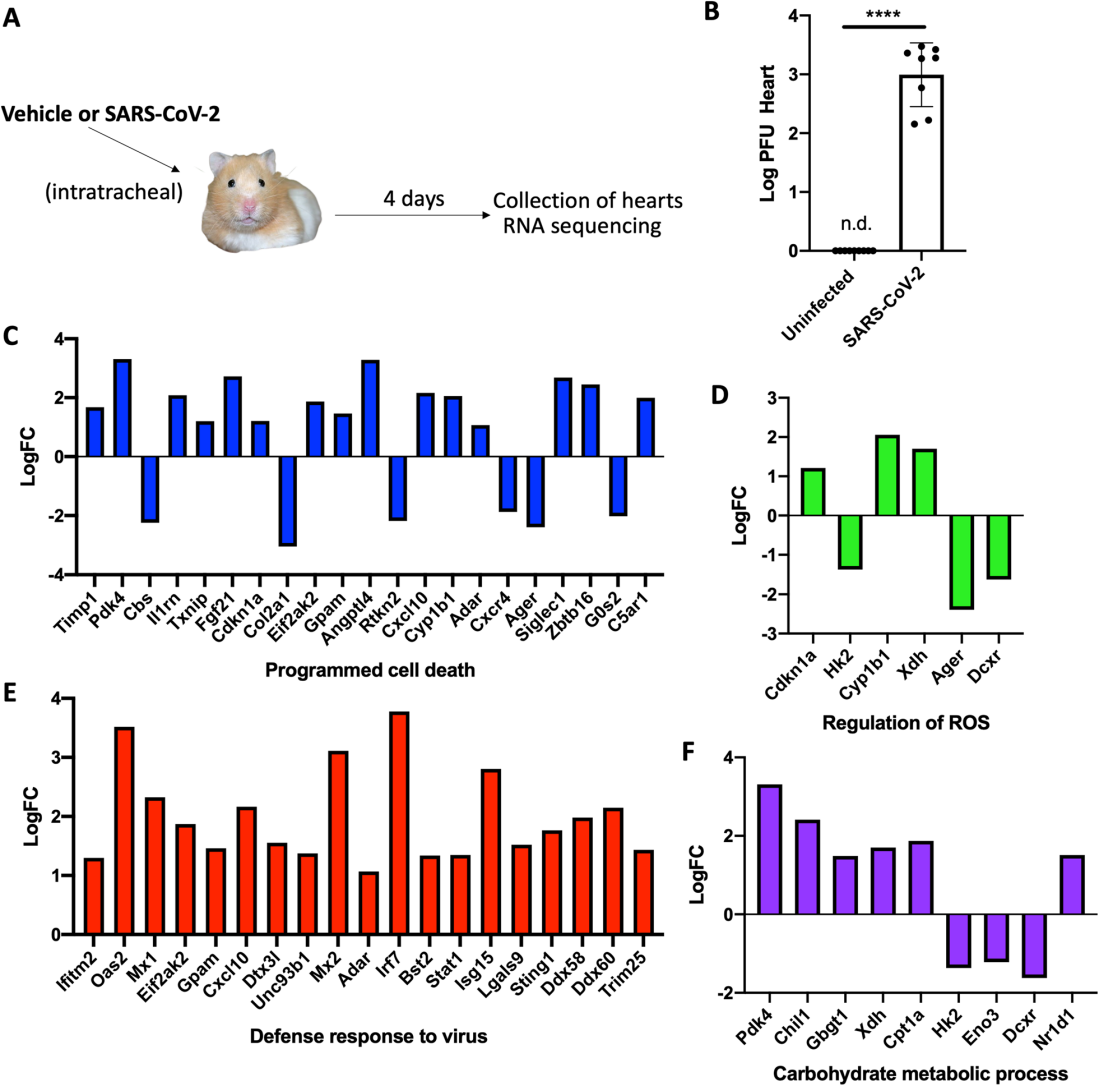
Cardiac transcriptional changes in a hamster model of severe SARS-CoV-2. **A** Male 5-to-6-week-old golden Syrian hamsters were IT infected with WA1, or mock challenged with vehicle (PBS), and hearts excised at 4 days post-infection for transcriptomics. **B** Log PFU per gram tissue of SARS-CoV-2 in hearts of hamsters (student *t*-test, *p-*value < 0.001 ****). **C–F** Log fold change (log FC) of transcriptional changes in infected hearts vs uninfected hamsters. Representative gene ontology terms selected were **C** programmed cell death, **D** regulation of ROS, **E** defence response to the virus, and **F** carbohydrate metabolic processes

To understand the underlying effects of severe SARS-CoV-2 infection in the heart, we used a recently established Golden Syrian Hamster model of severe infection [41]. Hamsters were infected intratracheally with SARS-CoV-2 strain USA-WA-1/2020 at a 9 X 10^5 PFU dose. Four days later, mice were sacrificed, and hearts were excised to assess viral titters and RNA sequencing (Fig. 4A). We observed a stark increase in viral titters (plaque forming units, PFU) in the hearts of hamsters 4 days post-infection when compared to mock-infected animals (Fig. 4B). At this time point, viral titters in the lungs were shown to start decreasing in a previous report by our group, using the same model [41]. Then, the cardiac transcriptome was evaluated by transcriptome sequencing (RNA-seq) of uninfected hearts and hearts infected with SARS-CoV-2 on day 4 post-infection. The infection resulted in the differential expression of 1,084 transcripts with a *p-*value of ≤ 0.05 out of 12,556 transcripts for a change of 8.63% of the total cardiac transcriptome. Gene ontology (biological processes) analysis for the significantly changed transcripts showed major changes in terms associated with programmed cell death (Fig. 4C), regulation of reactive oxygen species (ROS) (Fig. 4D), defence response to the virus (Fig. 4E), and carbohydrate metabolic processes (Fig. 4F). Of note, cell death and ROS activity in the heart have been linked to active pathogenesis of cardiovascular diseases [42] and cardiac damage in models of severe pneumococcal and pandemic influenza infections [15, 21, 23, 43]. The stark response to viral infection shown in the transcriptional increase of factors such as Ifitm2, Irf7, Stat1, and Eif2a, among others (Fig. 4E), indicates a heart actively mounting a host response to the invading SARS-CoV-2 (Fig. 4B).

### Hamsters and humans show the activity of programmed necrosis, i.e. necroptosis in the heart and serum, respectively

**Fig. 5:**
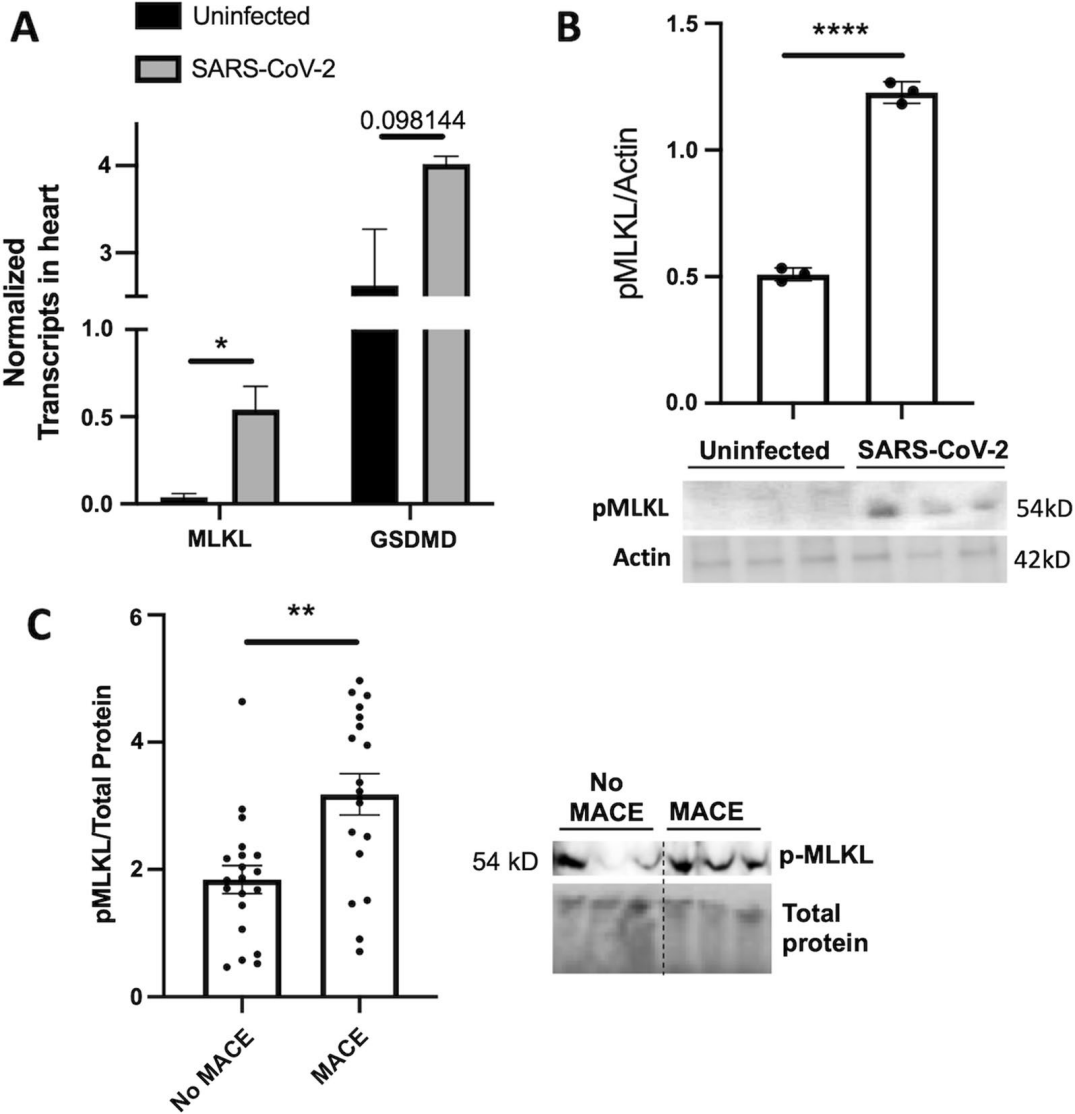
Hamster hearts show increased necroptosis in the model of severe SARS-CoV-2. **A–C** Male 5-to-6-week-old golden Syrian hamsters were IT infected with WA1 or mock challenged with vehicle (PBS) and hearts excised at 4 days post-infection for transcriptomics**. A** Normalised Transcript levels of MLKL and gasdermin-D (GSDMD) in uninfected vs SARS-CoV-2 infected hamsters. **B** Immunoblot for pMLKL in homogenates of hamsters' hearts. **C** Immunoblot for pMLKL in serum of hospitalised patients experiencing MACE or no-MACE. Mean ± SEM analysed by **B** student *T* test or **C** Kruskal–Wallis test with Dunn's multiple-comparison post-test. Asterisks denote the level of significance observed: * = *p* ≤ 0.05; ** = *p* ≤ 0.01; *** = *p* ≤ 0.001; *** = *p* ≤ 0.0001

Analysis of transcriptional changes of the effectors of the two major programmed necrosis pathways, necroptosis (mixed lineage kinase domain-like; MLKL) and pyroptosis (gasdermin D; GSDMD), showed necroptosis to be starkly elevated in SARS-CoV-2 infected hamsters compared to the hearts of the uninfected group (Fig. 5A). We used immunoblots to confirm whether these transcription changes reflected protein and activity levels. Blots for the phosphorylated MLKL (pMLKL), the active form of MLKL, in hamster hearts showed significant activity of necroptosis in the hearts of SARS-CoV-2-infected hamsters (Fig. 5B).

While hamsters provide a pivotal model to study SARS-CoV-2 cardiac pathogenesis, we aimed to define if such a strong indicator of tissue injury was present in our MACE-experiencing human cohort. Immunoblots for pMLKL showed a distinct increase in the presence of this molecular marker of necroptotic cell death in the serum of MACE patients compared to those without MACE (Fig. 5C). These results suggest necroptosis activation in patients with MACE, which correlates with the findings described in the animal model. The complete uncropped gel and blot images are in the Additional File 1: (Figure S1). Of note, no major changes in either GSDMD or the effector molecule of apoptosis, caspase-3, were observed between the MACE and No-MACE groups Additional File 1: (Figure S2).

### Markers of cardiac injury, inflammation, necroptosis, and circulating SARS-CoV-2 strongly correlate with the development of MACE

**Fig. 6:**
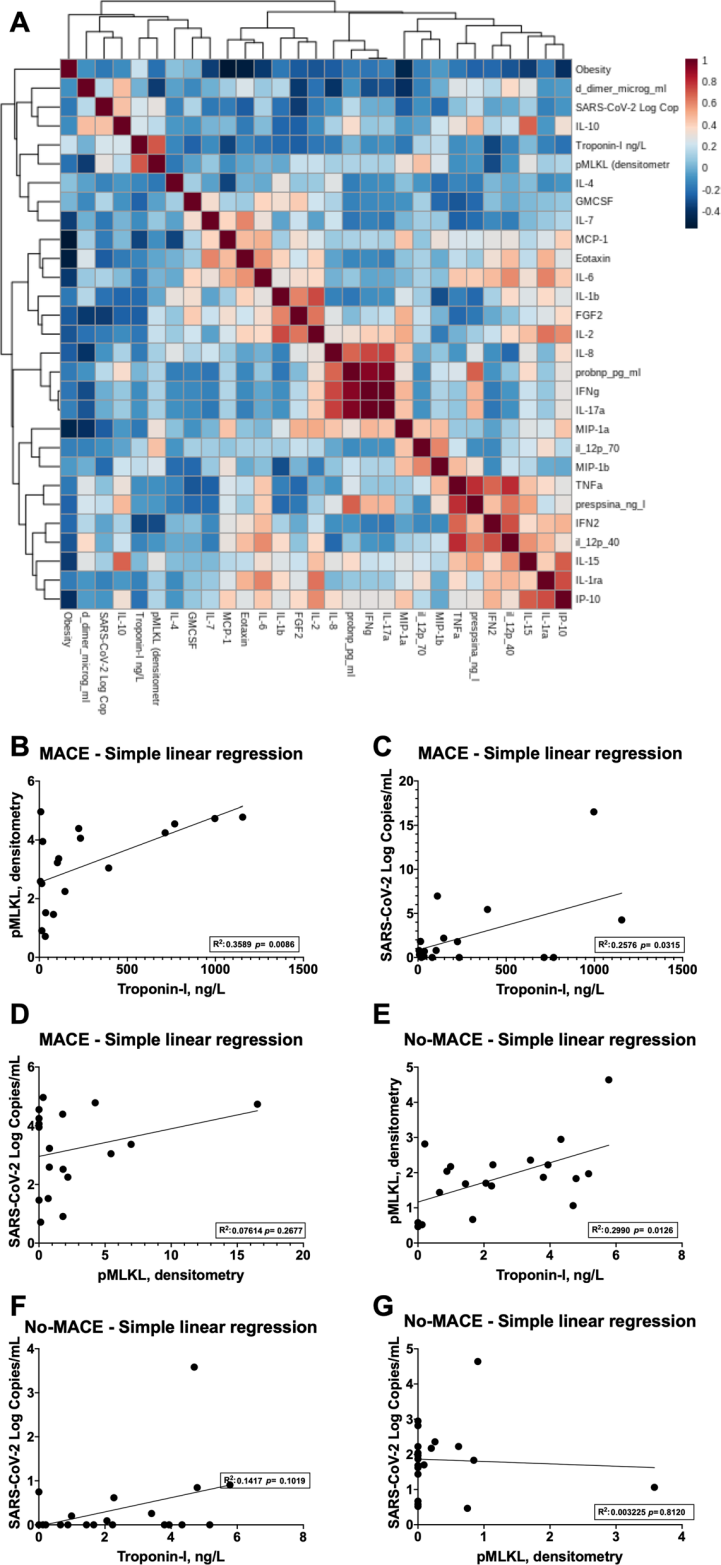
Markers of cardiac injury, inflammation, necroptosis, and SARS-CoV-2 correlate with MACE. **A** Correlation plot of serum cytokines, chemokines, biomarkers of cardiac injury, cell death effector MLKL, and SARS-CoV-2 (Log copies/mL). Linear regression plots for pMLKL—Troponin-I, SARS-CoV-2 (Log copies/mL)—Troponin-I and SARS-CoV-2 (Log copies/mL)—pMLKL (densitometry) in patients experiencing **B–D** MACE or **E–G** No-MACE

Using an annotated heatmap, we evaluated the association of MACE with all analysed serum biomarkers (Fig. 6A). We observed that Troponin-I and pMLKL were the main variables associated with the development of MACE in humans. Then, single linear regressions were performed to analyse these relations further. We found a correlation between high serum pMLKL and high serum Troponin-I levels with a regression coefficient of 0.3589, *p* = 0.0086 (Fig. 6B) in MACE patients. On top of that, in the no-MACE, the correlation was also significant due to low pMLKL and Troponin-I (Fig. 6E). Notably, the serum viral burden evaluated by SARS-CoV-2 copies was associated with Troponin-I release in patients with MACE (Fig. 6C), with a regression coefficient of 0.2576, *p* = 0.03, but not in no-MACE patients (Fig. 6F). SARS-CoV-2 copies and pMLKL levels in serum did not correlate with MACE (Fig. 6D) and no-MACE (Fig. 6G) groups. Of note, the top four biomarkers for the development of MACE were observed using a mean accuracy plot and defined to be pMLKL, Troponin-I, serum presence of SARS-CoV-2, and Pro-BNP (Additional File 1: Figure S3). These findings illustrate the relation between necroptosis, cardiac injury, and viral burden with the development of MACE.

## Discussion

In this comprehensive assessment of the underlying mechanisms of MACE, we found that SARS-CoV-2 can infect hamster heart tissue, propagate, and induce cardiac damage by activating necroptosis. We also observed that human participants with COVID-19 who developed MACE had a higher viral burden, necroptosis activity, and markers of cardiac injury (troponin and pro-BNP) in the serum. In this cohort, we did not detect any significant difference in the neutralising antibody breadth against SARS-CoV-2 variants. This observation suggests that antibody-neutralising activity is not associated with protection from MACE progression at admission. These findings are novel and constitute an important advance in understanding MACE in patients with COVID-19 and propose some potential therapeutic targets.

Several epidemiological studies have extensively characterised MACE during and after COVID-19 [7, 27, 44, 45]. A recent systematic review of 150 studies with 33,805 patients showed that COVID-19 increased the risk of developing MACE. These complications were more frequently documented in patients with pre-existing cardiovascular comorbidities and disease severity [46]. However, a recent report by Xie et al*.* showed that even mild cases of COVID-19 have a high risk of developing MACE, such as heart failure and stroke, 1-year after recovering from the disease. Cardiac dysfunction has substantial implications for the treatment of COVID-19 but also demands deep mechanistic research to aid in reducing acute and long-lasting disease sequela (*i.e*., long-COVID) [10]. Of note, using a rodent model of infection Dhanyalayam et al. observed that SARS-CoV-2 could persist in the cardiac tissue of male and female humanised ACE2 of infected mice (USA-WA1/2020) at 10 days post-infection. The SARS-CoV-2 infection led to sex-dependent morphological changes in the hearts of mice. Males were found more susceptible to pathological changes such as immune cell infiltration, and a slight increase in fibrosis was observed in both sexes [47]. However, the molecular and immune determinants that modulate such pathology are unknown. The results from the presented study propose that SARS-CoV-2 drives programmed necrosis as a mechanism for cardiac injury and cardiovascular complications associated with COVID-19. An increase in circulatory SARS-CoV-2 signal (DNA copies/mL) is associated with increased markers of cardiac injury (Troponin-I and pro-BNP) and the presence of necroptosis effector pMLKL in serum. To corroborate this observation, we infected golden Syrian hamsters with SARS-CoV-2 and detected an increase in virus titters in the heart and the activity of necroptosis via transcriptomics and immunoblots. Taken together, our results propose a new mechanism for SARS-CoV-2-driven cardiac injury via activation of necroptosis. It also provides a new therapeutic target to reduce the acute and long-term cardiac damage associated with COVID-19.

Most epidemiological and systematic reviews regarding MACE and COVID-19 have suggested Troponin-I increase as a major biomarker for MACE and prevalent disease outcomes, which was expected [5, 7, 32, 48, 49]. In this study, we found that viral burden and high concentrations of the necroptosis effector pMLKL in the serum were more accurate in predicting the development of MACE and possibly long-term cardiac events in convalescent COVID-19 patients. This is important as the MACE patients in this study had marginal changes in overall inflammatory circulating cytokines and chemokines associated with cell death and injury or in biomarkers of cardiac damage driven by inflammation (*i.e*., presepsin). Inflammation has been proposed as the primary driver of severe COVID-19 [50–52] and, in some cases, of MACE [50–52]. The currently available data support the requirement of future longitudinal studies aiming to define the role of pro-necroptosis cytokines and chemokines [53] in MACE development.

Necroptosis is a highly inflammatory form of cell death regulated by the receptor-interacting serine/threonine-protein kinases (RIPK)1 and RIPK3, and MLKL [54, 55]. Recently, a role for the parallel activation of multiple programmed cell death pathways (PANoptosis) due to a death signal has been implicated in disease pathogenesis [53, 56]. During viral infections, the differential activation of adenosine deaminase acting on RNA 1 (ADAR1) and Z-DNA binding protein 1 (ZBP1) regulate the activity of PANoptosis and innate immune responses [57]. Our transcriptomic data (Bio project ID–PRJNA884511) support the notion of differential changes in the expression of several adenosine deaminases (data not shown). However, additional mechanistic studies are required to understand the effects of adenosine deaminase downregulation in promoting MACE. Our study suggests that necroptosis can be a therapeutic target to reduce the acute and long-term effects of COVID-19 on the heart. Supporting this notion, our group has shown that inhibiting necroptosis or oxidative stress reduces cardiac injury observed during and after pneumococcal pneumonia [21]. Treatment with FDA-approved tyrosine kinase and necroptosis inhibitor "Ponatinib" (by inhibition of RIPK1 and RIPK3 [58]) was found to reduce collagen deposition, troponin release, and promote cardiac function up to 3 months after pneumococcal pneumonia when used as an adjunct therapy to antibiotics in a mouse model[21]. Notably, while beneficial during bacterial infection, Ponatinib blocks upstream effectors of necroptosis required in the cell defence against viruses [58, 59]; thus, future studies should aim to use selective MLKL inhibitors to reduce cardiac injury driven by SARS-CoV-2.

While this study represents a comprehensive approach to dissecting some mechanisms of MACE, it has several limitations. First, we could not study the heart tissue of patients that developed MACE as our group lacked access to these tissues. However, we performed a robust clinical and paraclinical characterisation of these patients that allowed us to identify necroptosis and viral burden as crucial factors for developing MACE. Second, the number of patients included in this study was small, which limits our capacity to develop high-accuracy predictive models using the serum biomarkers identified in our study. However, future studies will define how these markers directly correlate with MACE and if additional effectors can be linked to these phenotypes. Third, the kinetics of the study is not longitudinal; however, the data presented on Log copies/mL of SARS-CoV-2 were from samples collected upon admission to ICU. Thus, MACE patients showed more viral load persistence in serum.

In conclusion, the presented study suggests that necroptosis and circulating viral particles are primary drivers of MACE in severe COVID-19 patients. Notably, systemic inflammation and antibody responses to different viral variants were not associated with the development of MACE in humans. Herein we propose that pMLKL, Troponin-I, and pro-BNP can be more accurate biomarkers for acute and future development of MACE and disease outcomes. Finally, our data support the notion that inhibition of MLKL can be a possible new therapeutic or adjunct therapeutic approach to prevent the acute and long-term MACE caused by cardiac injury during severe COVID-19.

## Supplementary Information

**Additional file 1.** Complete uncropped gel and blot images of Hamsters Hearts and human serum. **A.** Immunoblot for pMLKL in homogenates of hamsters' hearts (Panel B in Fig. 5 of the original manuscript). **B.** Immunoblot for pMLKL in serum of hospitalized patients experiencing MACE or no-MACE (Panel C in Fig. 5 of the original manuscript).

**Additional file 2.** Pyroptosis and apoptosis are not overly active in patients with MACE. Immunoblot for Gasdermin-D (pro-[53 kDa], activated [30 kDa], and inactivated [20 kDa]) and Caspase-3 (pro-[35 kDa] and cleaved [19 kDa and 17 kDa]) in serum of hospitalized patients experiencing MACE or no-MACE. Uncropped gels are shown below.

## Data Availability

The datasets used and/or analysed during the current study are available from the corresponding author upon reasonable request.
