# Supplementary material for: Major adverse cardiovascular events are associated with necroptosis during severe COVID-19
Source: Crit Care. 2023 Apr 20;27:155. doi: 10.1186/s13054-023-04423-8 (PMC10116454; doi:10.1186/s13054-023-04423-8)
Supplement: Supplementary file 1 — Additional file 1. Complete uncropped gel and blot images of Hamsters Hearts and human serum. A. Immunoblot for pMLKL in homogenates of hamsters' hearts (Panel B in Fig. 5 of the original manuscript). B. Immunoblot for pMLKL in serum of hospitalized patients experiencing MACE or no-MACE (Panel C in Fig. 5 of the original manuscript). [file 13054_2023_4423_MOESM1_ESM.docx]

**TITLE:** Major Adverse Cardiovascular Events are Associated with Necroptosis During Severe COVID-19

**AUTHORS:** Rosana Wiscovitch-Russo^1#^, Ph.D.; Elsa D. Ibáñez-Prada^2,3#^, M.D.; Cristian C. Serrano-Mayorga^2,3^, M.D.; Benjamin L. Sievers^1^, Ph.D.; Maeve A. Engelbride^1^, Ph.D.; Surya Padmanabhan^1^, Ph.D.; Gene S. Tan^1,4^, Ph.D.; Sanjay Vashee^1^, Ph.D.; Ingrid G. Bustos^2^, Esp; Carlos Pachecho^2,3^, Esp; Lina Mendez^3^, MSc; Peter H. Dube^5$^, Ph.D.; Harinder Singh^1^, Ph.D.; Luis Felipe Reyes*^##2,3,6^, M.D., MSc, Ph.D.; and Norberto Gonzalez-Juarbe*^##1^, Ph.D.

**AFFILIATIONS:** ^1^Infectious Diseases and Genomic Medicine Group, J Craig Venter Institute, 9605 Medical Center Drive Suite 150, Rockville, MD, USA; ^2^Universidad de la Sabana, Chía, Colombia; ^3^Clinica Universidad de La Sabana, Chía, Colombia; ^4^Division of Infectious Diseases, Department of Medicine, University of California San Diego, La Jolla, CA 92037, USA; ^5^Department of Microbiology, Immunology and Molecular Genetics, The University of Texas Health Science Center at San Antonio, San Antonio, TX 78229, USA; ^6^Pandemic Science Institute, University of Oxford, Oxford, United Kingdom.

*** Corresponding authors:** Luis Felipe Reyes, MD., PhD., Universidad de La Sabana, Chía, Colombia. Phone number: 57 861 55 55 Ext: 23342. Email: [luis.reyes5@unisabana.edu.co](mailto:luis.reyes5@unisabana.edu.co), Norberto Gonzalez-Juarbe, PhD, Infectious Diseases and Genomic Medicine Group, J Craig Venter Institute, 9605 Medical Center Drive Suite 150, Rockville, MD 20850, USA. Tel. 795-301-7393, Fax. 301-795-7051, Email: [ngonzale@jcvi.org](mailto:ngonzale@jcvi.org)

**# Co-first Authors, ## Co-Senior Authors**

**$ Current Address:** Boehringer Ingelheim, Ames, Iowa, United States.

**ADDITIONAL FIGURES**

**Additional Figure S1: Complete uncropped gel and blot images of Hamsters Hearts and human serum. A.** Immunoblot for pMLKL in homogenates of hamsters' hearts (Panel B in Figure 5 of the original manuscript). **B.** Immunoblot for pMLKL in serum of hospitalized patients experiencing MACE or no-MACE (Panel C in Figure 5 of the original manuscript).

**Additional Figure S2: Pyroptosis and apoptosis are not overly active in patients with MACE.** Immunoblot for Gasdermin-D (pro-[53 kDa], activated [30 kDa], and inactivated [20 kDa]) and Caspase-3 (pro-[35 kDa] and cleaved [19 kDa and 17 kDa]) in serum of hospitalized patients experiencing MACE or no-MACE. Uncropped gels are shown below.

**
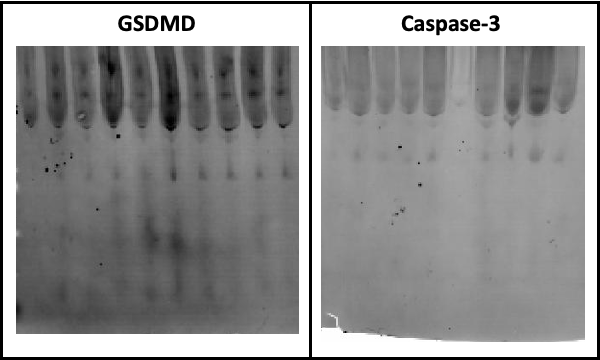
**


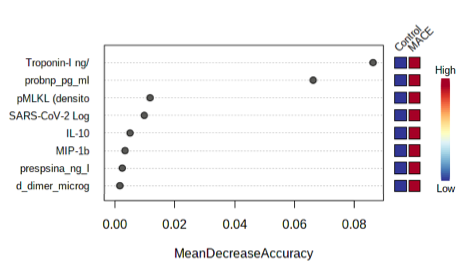


**Additional Figure S3: Troponin-I, pro-BNP, pMLKL, IL-10, and circulating SARS-CoV-2 are robust prognostic biomarkers of MACE**. The mean Accuracy plot shows the accuracy of specific biomarkers to define the development of MACE in human patients with COVID-19.
