# Supplementary material for: Major adverse cardiovascular events are associated with necroptosis during severe COVID-19
Source: Crit Care. 2023 Apr 20;27:155. doi: 10.1186/s13054-023-04423-8 (PMC10116454; doi:10.1186/s13054-023-04423-8)
Supplement: Supplementary file 2 — Additional file 2. Pyroptosis and apoptosis are not overly active in patients with MACE. Immunoblot for Gasdermin-D (pro-[53 kDa], activated [30 kDa], and inactivated [20 kDa]) and Caspase-3 (pro-[35 kDa] and cleaved [19 kDa and 17 kDa]) in serum of hospitalized patients experiencing MACE or no-MACE. Uncropped gels are shown below. [file 13054_2023_4423_MOESM2_ESM.docx]

**TITLE:** Major Adverse Cardiovascular Events are Associated with Necroptosis During Severe COVID-19

**AUTHORS:** Rosana Wiscovitch-Russo^1#^, Ph.D.; Elsa D. Ibáñez-Prada^2,3#^, M.D.; Cristian C. Serrano-Mayorga^2,3^, M.D.; Benjamin L. Sievers^1^, Ph.D.; Maeve A. Engelbride^1^, Ph.D.; Surya Padmanabhan^1^, Ph.D.; Gene S. Tan^1,4^, Ph.D.; Sanjay Vashee^1^, Ph.D.; Ingrid G. Bustos^2^, Esp; Carlos Pachecho^2,3^, Esp; Lina Mendez^3^, MSc; Peter H. Dube^5$^, Ph.D.; Harinder Singh^1^, Ph.D.; Luis Felipe Reyes*^##2,3,6^, M.D., MSc, Ph.D.; and Norberto Gonzalez-Juarbe*^##1^, Ph.D.

**AFFILIATIONS:** ^1^Infectious Diseases and Genomic Medicine Group, J Craig Venter Institute, 9605 Medical Center Drive Suite 150, Rockville, MD, USA; ^2^Universidad de la Sabana, Chía, Colombia; ^3^Clinica Universidad de La Sabana, Chía, Colombia; ^4^Division of Infectious Diseases, Department of Medicine, University of California San Diego, La Jolla, CA 92037; ^5^Department of Microbiology, Immunology and Molecular Genetics, The University of Texas Health Science Center at San Antonio, San Antonio, TX 78229, USA; ^6^Pandemic Science Institute, University of Oxford, Oxford, United Kingdom.

*** Corresponding authors:** Luis Felipe Reyes, MD., PhD., Universidad de La Sabana, Chía, Colombia. Phone number: 57 861 55 55 Ext: 23342. Email: [luis.reyes5@unisabana.edu.co](mailto:luis.reyes5@unisabana.edu.co), Norberto Gonzalez-Juarbe, Ph.D., Infectious Diseases and Genomic Medicine Group, J Craig Venter Institute, 9605 Medical Center Drive Suite 150, Rockville, MD 20850. Tel. 795-301-7393, Fax. 301-795-7051, Email: [ngonzale@jcvi.org](mailto:ngonzale@jcvi.org)

**# Co-first Authors, ## Co-Senior Authors**

**$ Current Address:** Boehringer Ingelheim, Ames, Iowa, United States.

**METHODS**

*Quantification of cytokines and chemokines*

Venous blood was collected using EDTA tubes within the first 24 hours of hospital admission and was centrifuged at 1000 × g for 10 minutes within 30 minutes. The plasma was removed and frozen at −80°C in aliquots until cytokines analysis. Before analysis with Multiplex (Luminex) cytokine assays, samples were thawed completely, mixed, and centrifuged. The analysis was conducted using 25 μL of plasma sample. The measured cytokines were basic fibroblast growth factor, Eotaxin, granulocyte-macrophage colony-stimulating factor, interferon α-2, interferon-γ, IL-10, IL-15, IL-1α, IL-6, IL-8, IP-10, monocyte chemoattractant protein-1, macrophage inflammatory protein-1β, and tumor necrosis factor-α. Results were determined by standard curve analysis, and plasma concentrations were determined with the Human Cytokine/Chemokine Magnetic Bead Panel kit from Millipore (HCYTOMAG-60K) (Merck).

Antibody-bead vials were sonicated for 30 seconds and vortexed for 1 min afterward. Then, 60 µL of each antibody bead vial was added to the mixing bottle, followed by 1.68 mL of bead diluents to make the final volume 3.0 mL. The standards, quality controls, and serum matrix were reconstituted according to the manufacturer's instructions. The standards were serially diluted 1 to 5 [0 (Background), 3.2, 16, 80, 400, 2.000, and 10.000 pg per mL] in an assay buffer. The latter was used for background wells, and one quality control was also included in the study. Samples placed in the 96-well plates were read and analyzed on the MAGPIX® System instrument using xPONENT® Software version 4.2. Standard curves were drawn for each cytokine, and concentrations were determined from the standard curve using a 5-point regression to transform the median fluorescence intensity values into concentrations for each analyte evaluated. Any value below the detection level was replaced by the limit of detection (LOD), as reported by the Luminex kit.

*RNA sequencing*

Total RNA was isolated from the hearts of hamsters infected with SARS-CoV-2 or mock-treated with vehicles. Hearts were homogenized and placed in TRIzol. Bulk RNA in TRIzol was purified according to the manufacturer's instructions. RNA was further purified using the RNeasy kit (Qiagen). Library preparation and RNA-seq were performed by the Sequencing Core at the J. Craig Venter Institute. The RSEM package and Bowtie2 were used for quantifying gene and isoform abundances from the paired-end RNA-seq data ^32^. Differential gene expression analysis was performed using the edgeR package ^33^.

*Neutralization assays*

Neutralization assays were performed as previously described ^34^. Briefly, Vero E6-TMPRSS2-T2A-ACE2 (BEI Resources) were seeded at 5 × 10^5^ cells per well 24 hours before performing the neutralization assay. Plasma was serially diluted five-fold in media in duplicate. Twenty-five microliters of a VSV encoding enhanced GFP gene pseudotyped with one spike protein variant and a second VSV encoding a mCherry gene pseudotyped with another spike protein variant. Then, the pseudotyped particles and plasma mixture were transferred onto the cell monolayer and incubated at 37°C and 5% CO2 for 24 hours. Finally, the number of GFP- and RFP-expressing cells were quantified using a Celigo Image Cytometer (Nexcelcom Bioscience). We then calculated the percent infection and the percent inhibition.

*Western Blots*

Samples were loaded into 12.5% gradient gels at 20 μg per lane, separated by SDS-PAGE, and transferred to nitrocellulose membranes. Total protein was quantified by Ponceau stain, and then membranes were blocked using Tris-buffered saline [TBS]-0.01% Tween 20 containing 5% bovine serum albumin [BSA] for at least 1h. The primary antibody against pMLKL (91689, Cell Signaling Technology) was diluted in blocking buffer, and membranes were incubated overnight at 4°C. HRP-tagged secondary antibody was used to detect the primary antibody and detected (developed) using SuperSignal West PICO Plus (ThermoFisher 34580). All images were collected on an Amersham Imager 680 (GE) and analyzed for densitometry in ImageJ as previously described ^35^.
